# AcornHRD: an HRD algorithm highly associated with anthracycline-based neoadjuvant chemotherapy in breast cancer in China

**DOI:** 10.1186/s40001-024-01936-y

**Published:** 2024-07-16

**Authors:** Jia-Ni Pan, Pu-Chun Li, Meng Wang, Ming-Wei Li, Xiao-Wen Ding, Tao Zhou, Hui-Na Wang, Yun-Kai Wang, Li-Bin Chen, Rong Wang, Wei-Wu Ye, Wei-Zhu Wu, Feng Lou, Xiao-Jia Wang, Wen-Ming Cao

**Affiliations:** 1 Department of Breast Medical Oncology, Zhejiang Cancer Hospital, Hangzhou, 310022 China; 2 Institute of Basic Medicine and Cancer (IBMC), Chinese Academy of Sciences, Hangzhou, 310018 China; 3 Cancer Centre, Faculty of Health Sciences, University of Macau, Macau, 999078 SAR China; 4 Wenzhou Medical University, Wenzhou, 325035 China; 5 AcornMed Biotechnology Co., Ltd., Floor 18, Block 5, Yard 18, Kechuang 13 RD, Beijing, 100176 China; 6 Department of Breast Surgery, Zhejiang Cancer Hospital, Hangzhou, 310022 China; 7 Lihuili Hospital of Ningbo Medical Center, Ningbo, 315040 China

## Abstract

**Purpose:**

Our study aimed to develop and validate a homologous recombination deficiency (HRD) scoring algorithm in the Chinese breast cancer population.

**Methods and materials:**

Ninety-six in-house breast cancer (BC) samples and 6 HRD-positive standard cells were analyzed by whole-genome sequencing (WGS). Besides, 122 BCs from the TCGA database were down-sampled to ~ 1X WGS. We constructed an algorithm named AcornHRD for HRD score calculated based on WGS at low coverage as input data to estimate large-scale copy number alteration (LCNA) events on the genome. A clinical cohort of 50 BCs (15 cases carrying *BRCA* mutation) was used to assess the association between HRD status and anthracyclines-based neoadjuvant treatment outcomes.

**Results:**

A 100-kb window was defined as the optimal size using 41 in-house cases and the TCGA dataset. HRD score high threshold was determined as HRD score ≥ 10 using 55 in-house BCs with *BRCA* mutation to achieve a 95% *BRCA*-positive agreement rate. Furthermore, the HRD status agreement rate of AcornHRD is 100%, while the ShallowHRD is 60% in standard cells. *BRCA* mutation was significantly associated with a high HRD score evaluated by AcornHRD and ShallowHRD (*p* = 0.008 and *p* = 0.003, respectively) in the TCGA dataset. However, AcornHRD showed a higher positive agreement rate than did the ShallowHRD algorithm (70% vs 60%). In addition, the *BRCA-*positive agreement rate of AcornHRD was superior to that of ShallowHRD (87% vs 13%) in the clinical cohort. Importantly, the high HRD score assessed by AcornHRD was significantly correlated with a residual cancer burden score of 0 or 1 (RCB0/1). Besides, the HRD-positive group was more likely to respond to anthracycline-based chemotherapy than the HRD-negative group (pCR [OR = 9.5, 95% CI 1.11–81.5, *p* = 0.040] and RCB0/1 [OR = 10.29, 95% CI 2.02–52.36, *p* = 0.005]).

**Conclusion:**

Using the AcornHRD algorithm evaluation, our analysis demonstrated the high performance of the LCNA genomic signature for HRD detection in breast cancers.

**Supplementary Information:**

The online version contains supplementary material available at 10.1186/s40001-024-01936-y.

## Introduction

Breast cancer susceptibility genes *BRCA1* and *BRCA2* are involved in homologous recombination (HR) and play a pivotal role in the repair of DNA double-strand breaks [1]. Cancers with loss of HR function due to the inactivation of *BRCA1/2* and other HRR genes are known to be sensitive to platinum and poly (adenosine diphosphate-ribose) polymerase (PARP) inhibitors [2–4]. Germline *BRCA* mutations account for 5.3% of all breast cancers [5], and Turner [6] showed that homologous recombination deficiency (HRD) has a prevalence of approximately 18% in breast cancer. Thus, HRD testing would allow more precise treatment recommendations and provide benefits to populations receiving platinum and PARP inhibitors (PARPi). Moreover, the conclusion has been confirmed in multiple clinical trials of ovarian cancer. Patients with *BRCA* wild-type but positive HRD have an equal benefit from PARPi compared with *BRCA* mutations, based on the results of the PRIMA study and the PAOLA-1 study [7, 8]. While commercial HRD detection methods are available abroad, there is no uniform standard in China so far.

In current practice, anthracycline-based regimens and the sequential administration of taxanes are the most commonly used chemotherapy regimens in neoadjuvant and adjuvant settings. Prior studies have shown that platinum chemotherapy agents are active in the treatment of breast cancer with a germline *BRCA* mutation and/or HRD [9–11]. In the neoadjuvant setting, a single-arm prospective study using cisplatin monotherapy reported a pathologic complete response (pCR) rate of 61% among *BRCA1*-mutated breast cancer patients [12]. Moreover, the GeparSixto trial demonstrated that the pCR rates were 33.9% and 63.5% in the paclitaxel plus liposomal doxorubicin (PM) group and PM plus carboplatin group, respectively, among HRD breast cancer patients [11]. Conversely, the INFORM trial results showed that anthracycline-based regimens are also effective in HER2-negative *BRCA*-mutated breast cancer. The pCR rate was 18% and 26% in the single-agent cisplatin group and doxorubicin-cyclophosphamide group, respectively, which yielded a risk ratio (RR) of 0.70 (90% CI 0.39–1.2) [13]. Moreover, it was recently reported that breast cancers with high HRD scores are more sensitive to anthracycline in the neoadjuvant setting [14, 15].

This study aimed to develop an HRD scoring algorithm based on the Chinese population and compare its performance with the Shallow algorithm in different cohorts. To validate the accuracy of this HRD scoring algorithm, we evaluated the correlation between HRD scores with *BRCA* mutations and pCR for anthracycline-based neoadjuvant chemotherapy (NAC).

## Methods and materials

### DNA extractions, library preparation, and sequencing

Five HRD-positive and 1 HRD-negative standards (Cat No. CBP90023) stored at -20℃ from Nanjing Cobioer Biosciences CO., LTD prepared for genome-wide DNA extraction. Tumor tissue was collected from 41 in-house samples (cohort I) and 55 in-house samples with *BRCA* mutations (cohort II) from 85 breast cancer patients, and leukocytes were collected from 50 baseline healthy control samples. All genomic DNA (gDNA) was extracted using the Genomic DNA Extraction Kit (Item No. DP304). According to the quantitative results of the QUIBT tool, 200 ng gDNA was used for library construction. Subsequently, 200 ng gDNA for each sample was transferred to a 50-μL Covaris tube and segmented to the main peak of 300–350 bp using the Covaris M220 instrument. Next, segmented DNA was end-repaired, A-tailed, and ligated with custom adapters in reaction pooling. The ligation product was amplified (6 cycles) and purified using AmpureXP beads (Agencourt/Beckman Coulter). After purification, the library was quantified using a Qubit 4 fluorimeter and the Qubit dsDNA HS Assay Kit (ThermoFisher). Finally, library fragment quality control was performed using Agilent 2100 Bioanalyzer and Agilent 2100 DNA 1000 Kit. Each library was programmed to generate ~ 3.5 Gb bases.

### Filter and variant calling

The FASTP tool [16] was applied for FASTQ file quality control to remove reads with the adaptor, low-quality bases. High-quality reads were aligned into the human genome (hg19) with Burrows-Wheeler Aligner [17]. Duplicate reads generated by PCR were marked using Picard (broadinstitute.github.io/picard/). Moreover, local realignment around known InDels and base quality were recalibrated, and duplicate reads were subsequently removed using the Sentieon tool [18]. Finally, base alternatives and InDels detected by Sentieon were annotated using Annovar [19]. A series of 122 aligned bam files (Supplementary Table 1) downloaded from the TCGA breast cancer database (www.cancer.gov/about-nci/organization/ccg/research/structural-genomics/tcga) were down-sampled to ~ 1X whole-genome sequencing (WGS) with SamBamba software [20]. Subsequently, all bam files were also processed with the above pipelines. *BRCA*-positive status implies that any mutation was detected in either *BRCA1* or *BRCA2* for each sample. Identified mutation criteria in BRCA were as follows: (1) mutation information was collected from the TCGA database for somatic mutation; and (2) for germline mutation identified by the in-house analysis pipeline, its status was annotated as likely pathogenic or pathogenic by either InterVar [21] or ClinVar [22], and the number of supporting allele reads was greater than 3.

### Workflow of the algorithm for HRD evaluation

We developed an internal algorithm for assessing the HRD status of cancer patients by detecting copy number variations (CNV) based on low-coverage WGS, termed AcornHRD. HRD score was predicted based on the count of large-scale copy number alteration (LCNA) events, and the methodology was similar to the LST (large-scale state transition) in SNP arrays. LCNA events were assessed by coverage of sliding windows in the genome. Here, windows (specific-sized regions) are continuous segments of the genome used to detect LCNA; the coverage refers to the number of reads in each specific position or window in WGS data. The detailed algorithm description can be divided into two parts. Part one was to calculate the CNV ratio in the window unit along the genome as follows:

i The coverage from WGS data were counted and normalized according to the library size and GC content with LOWESS [23] statistics to calculate a $Rati{o}^{gc-correction}$ in each window.
ii $Rati{o}_{j}^{M}$ calculated the median of the GC-corrected ratio for *j-*th window in the baseline samples. It is defined for a given window j as:
  $${\text{Ratio}}_{\text{j}}^{\text{M}}= {\text{Median}}\left({\text{Ratio}}_{\text{j}1}^{\text{gc}-\text{correction}}, {\text{Ratio}}_{\text{j}2}^{\text{gc}-\text{correction}}, \dots , {\text{Ratio}}_{\text{jn}}^{\text{gc}-\text{correction}}\right)$$
  *n is the number of samples in the baseline.
iii $Rati{o}_{j}^{gc-correction}$ represented the GC-corrected ratio for the *j-*th window of the test sample. The CNV ratio quantifies the relative abundance of a window compared to baseline samples. It is defined for a given window j as:
  $$\text{CNV Ratio}=\frac{Rati{o}_{j}^{gc-correction}}{Rati{o}_{j}^{M}}.$$

The CNV ratio result file was used as input data to estimate the HRD status for each sample. Part two was to detect the HRD status as follows: firstly, to minimize the impacts from highly complex genomic regions (such as centromere regions, telomere regions, and highly repetitive regions) and sex chromosomes, overlap windows were removed. Subsequently, CNV ratios in each window were processed with log_2_ fold change. Next, windows were merged into large segments with chromosome arm information and processed CNV ratio by the circular binary segmentation (CBS) method with the R procedure (bioconductor.org/packages/release/bioc/html/DNAcopy.html). Finally, the above segment larger than 10 Mb was defined as an LCNA event [24–26]. We calculated the HRD score, defined as the count of LCNA, which is determined by the coverage of 100-kb windows. Tumors with HRD scores ≥ 10 were classified as exhibiting HRD scores high (refer to the Results section for further details).

### Validation by clinical breast *cancer* samples

**Fig. 1:**
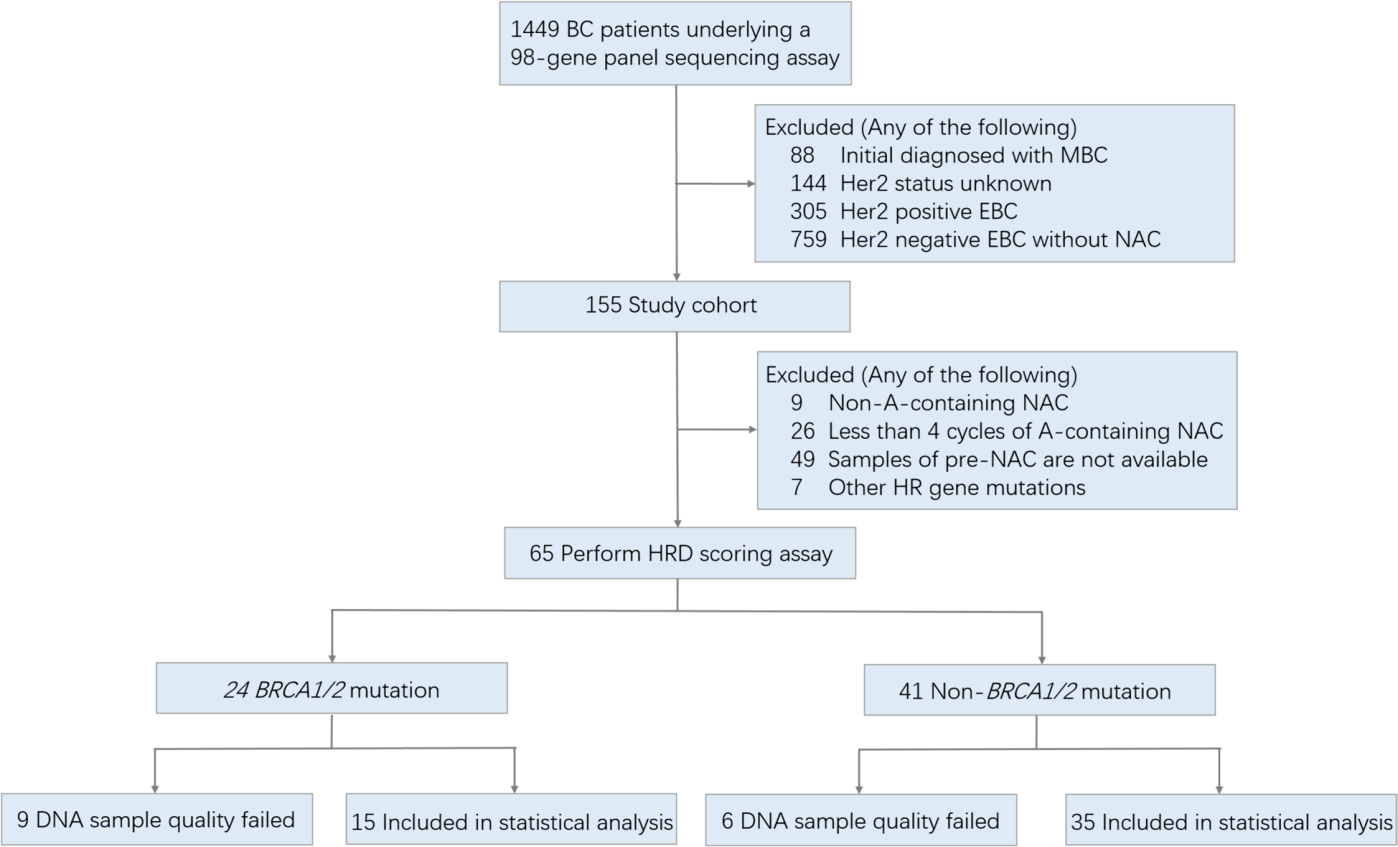
Patient selection criteria. Some patients met multiple exclusion criteria. BC: breast cancer, MBC: metastatic breast cancer, EBC: early breast cancer, A: anthracycline

We retrospectively reviewed the medical records of 1449 patients with primary breast cancer who visited the Zhejiang Cancer Hospital from February 2008 to October 2020 and completed a 98-gene panel genetic screening. Fifty patients who received anthracycline-based NAC and underwent subsequent surgery (mastectomy or breast-conserving surgery) were included in the statistical analysis (see Fig. 1 and Supplementary Table 2 for details). All of them received NAC with epirubicin (75 mg/m2) and cyclophosphamide (600 mg/m2), followed by docetaxel (80–100 mg/m2) every 3 weeks for 8 cycles. All biopsied tumor samples for histological and HRD examination were obtained from patients before NAC and kept by fixed-formalin paraffin-embedded (FFPE). The study was reviewed and approved by the Ethical Committee of Zhejiang Cancer Hospital and was performed in accordance with the Declaration of Helsinki.

### Statistical analyses

The primary endpoint was RCB 0/1 with a secondary endpoint of pCR. Fisher’s exact or Chi-squared test was used to test association with binary response and association of clinical variables and HRD score. We used the odds ratio (OR) as a measure of the strength of association between two variables and calculated a 95% confidence interval. A *P*-value less than 0.05 was considered statistically significant. All statistical analyses were performed using Python software version 3.

## Results

### Development of the AcornHRD algorithm

**Fig. 2:**
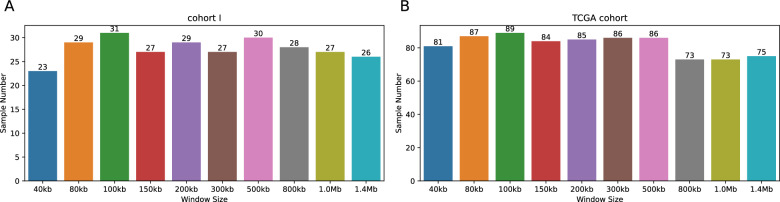
Mode frequency across ten distinct window sizes: For each sample, the mode of LCNA count is classified as 1, while other values are designated as 0. Subsequently, the frequency of mode samples is computed for each window. The horizontal axis signifies ten distinct window sizes, and the vertical axis denotes the sample count. Panel **A** shows the mode frequency in ten different window sizes for 41 breast cancer samples sourced from our in-house breast cancer cohort. Panel **B** displays the same for 122 breast cancer samples obtained from the TCGA database

AcornHRD was based on the results of the CNV ratio as input data to estimate LCNA events on the genome. A fitness window size appears particularly important for samples of low coverage. To address the question of optimal window size, we adopted up to ten different window sizes (40 kb, 80 kb, 100 kb, 150 kb, 200 kb, 300 kb, 500 kb, 800 kb, 1 Mb, and 1.4 Mb) to estimate the number of LCNA in each sample from cohort I (Supplementary Table 3). The results showed that the 100-kb window size covered most of the samples (31, 75.6%), followed by 500-kb window sizes (73.2%) (Fig. 2A). Furthermore, similar results were observed in the TCGA cohort, with the 100-kb window size still covering the largest number of samples (Fig. 2B). Additionally, this window size maintains a balance between capturing sufficient genomic information and minimizing noise, ensuring accurate LCNA identification. In summary, our findings reveal that although some alternative window sizes captured comparable percentages of samples, the 100-kb window size consistently exhibited the highest stability and resolution across different datasets.

### Establishing a threshold for the HRD score

HRD, another tumor biomarker, is being used in guiding therapy in an increasing number of studies [27–30]. Both deleterious mutations and promoter methylation of *BRCA1/2* could cause HRD and genomic instability. Furthermore, *BRCA* mutations are known to be strongly associated with HRD. The HRD threshold was selected to have a high sensitivity for detecting HR deficiency in breast cancer. We defined the *BRCA*-positive agreement rate as the proportion of the number of samples with a high HRD score in the *BRCA*-positive samples; the *BRCA*-negative agreement rate was the same as that mentioned above. To obtain a *BRCA*-positive agreement rate of at least 95%, the threshold was set at the 5th percentile of the HRD scores in cohort II (Supplementary Table 4) of known *BRCA*-positive tumors. The HRD scores of patients with *BRCA* mutation were assessed in the test panel with a 100-kb window size and 50-kb step size. Of 55 patients, 53 (96.4%) were identified as having a high HRD score due to a score greater than or equal to 10 (Supplementary Table 5). For a 95% confidence detection rate, the score of 10 was defined as the cut-off threshold value.

### Comparing HRD status with AcornHRD and ShallowHRD algorithms in the standard sample

For a more comprehensive evaluation of the AcornHRD algorithm, ShallowHRD software [31] was added to the following comparative analysis. As described in the method section, six standard cells were sequenced with whole genomic DNA. Compared to the HRD status of 6 standard samples (5 HRD positive and 1 HRD negative), the HRD status agreement rate of AcornHRD is 100%, while the agreement rate of ShallowHRD is only 60% (Table 1).

**Table 1:** HRD status of six standards assessed by AcornHRD and ShallowHRD

Standard ID		AcornHRD		ShallowHRD	
	Proven status	HRD score	HRD status	HRD score	HRD status
102109017T3	Positive	30	High	34	High
102109018T3	Positive	14	High	13	Low
102109019T3	Positive	25	High	22	High
102109020T3	Positive	12	High	12	Low
102109021T3	Positive	11	High	8	Low
102109022T3	Negative	3	Low	3	Low

Positive represents HRD standard sample and negative represents non-HRD standard sample

High: HRD score ≥ 10, Low: HRD score < 10 for AcornHRD

High: HRD score ≥ 15, Low: HRD score < 15 for ShallowHRD

### Correlation between BRCA mutations and HRD status with AcornHRD and ShallowHRD algorithms in the TCGA cohort

Mutations in the *BRCA* are strongly associated with HRD positivity [32, 33]; thus, we applied the *BRCA*-positive agreement rate to assess and compare the accuracy of the two HRD assessment methods in a large TCGA cohort (2 samples without somatic mutation information were filtered out) [31]. The mutations of *BRCA* genes were confirmed in tumor sequencing reads by in-house calling variation pipeline (more details presented in the Methods). Of the 120 patients, 20 (16.7%) harbored *BRCA* mutations (Supplementary Table 9). The results of AcornHRD (Table 2) and ShallowHRD (Table 3) both showed that *BRCA* mutation is significantly correlated with a high HRD score (p = 0.008 and p = 0.003, respectively). However, the *BRCA*-positive agreement rate of AcornHRD was higher than that of the ShallowHRD algorithm, which was 70% (14/20) and 60% (12/20), respectively (Tables 2 and 3).

**Table 2:** The HRD status according to *BRCA* mutations by AcornHRD in TCGA cohort

HRD status	*BRCA* status		Total
	Mutated	Non-mutated	
HRD score high	14 (70.0%)	38 (38.0%)	52
HRD score low	6 (30.0%)	62 (62.0%)	78
Total	20	100	120

HRD score high: HRD score ≥ 10; HRD score low: HRD score < 10

**Table 3:** The HRD status according to *BRCA* mutations by ShallowHRD in TCGA cohort

HRD Status	*BRCA* status		Total
	Mutated	Non-mutated	
HRD score high	12 (60.0%)	26 (26.0%)	38
HRD score low	8 (40.0%)	74 (74.0%)	82
Total	20	100	120

HRD score high: HRD score ≥ 15; HRD score low: HRD score < 15

### Association of HRD scores with responses to NAC by AcornHRD and ShallowHRD algorithms

Of the clinical cohort, 15 *BRCA*-positive and 35 *BRCA*-negative patients were considered to evaluate HRD status (Supplementary Table 6). The HRD score result showed that the *BRCA*-positive agreement rate of AcornHRD is far superior to that of ShallowHRD, whereas the *BRCA*-negative agreement rate is not as good (Supplementary Table 7 and Supplementary Table 8). In addition, the AcornHRD evaluation performed better than did that of the ShallowHRD score; specifically, the high HRD score was significantly associated with residual tumor burden 0/1 (*p* = 0.020 for AcornHRD and *p* = 0.182 for ShallowHRD) (Table 4). In summary, AcornHRD was more stable in the application performance across three different cohorts of WGS data, which is superior to the published algorithm.

**Table 4:** Association of HRD status with RCB from clinical cohort between AcornHRD and ShallowHRD

Clinical cohort (n = 50)	RCB0/1 *n* (%)	RCB2/3 *n* (%)	OR	95% CI	*P* value
Acorn HRD					
HRD score low	4 (18)	18 (82)	1		
HRD score high	14 (50)	14 (50)	4.5	1.21–16.72	0.020
Shallow HRD					
HRD score low	17 (41)	24 (59)	1		
HRD score high	1 (11)	8 (89)	0.18	0.02–1.55	0.182

HRD score high: HRD score ≥ 10, HRD score low: HRD score < 10 for AcornHRD

HRD score high: HRD score ≥ 15, HRD score low: HRD score < 15 for ShallowHRD

### Clinicopathologic characteristics of high HRD score tumors

Among the 50 patients in the clinical cohorts who received anthracycline-based neoadjuvant therapy, 28 had high HRD scores, and 22 had low HRD scores. A high HRD score was significantly correlated with *BRCA* mutations (see Table 5 and Supplementary Table 10 for details). The breast cancer samples selected for the clinical study were all *HER-2* negative, including 24 TNBC samples and 26 ER and/or PR-positive samples. A high HRD score significantly correlated with TNBC and high Ki-67 expression (Table 5). High HRD scores showed a trend toward correlation with ER-negative and PR-negative status (Table 5).

**Table 5:** Patient characteristics and HRD score from clinical cohort

	HRD score high (*n* = 28)	HRD score low (*n* = 22)	OR	95% CI	*P* value
Age				0.58–5.62	0.310
> 40 years	10	11	1		
≤ 40 years	18	11	1.8		
BMI(kg/m^2)				0.54–6.67	0.320
< 25	18	17	1		
≥ 25	10	5	1.89		
*BRCA* status				1.69–44.34	0.011
Non-mutated	15	20	1		
Mutated	13	2	8.67		
Menopause				0.11–2.72	0.730
Pre-	25	18	1		
Post-	3	4	0.54		
ER				0.10–1.02	0.050
Negative	18	8	1		
Positive	10	14	0.32		
PR				0.10–1.05	0.057
Negative	19	9	1		
Positive	9	13	0.33		
Ki-67				1.33–25.05	0.032
< 20%	3	9	1		
≥ 20%	25	13	5.77		
Molecular subtype				1.02–10.72	0.042
Non-TNBC	11	15	1		
TNBC	17	7	3.31		

HRD score high: HRD score ≥ 10; HRD score low: HRD score < 10 for AcornHRD

OR: odds ratio, CI: confidence interval, Pre-: premenopause, Post-: postmenopause

### Correlation between HRD status and NAC efficacy

In this study, HRD positivity includes either a high HRD score or a *BRCA* mutation, whereas HRD negativity included a low HRD score and no *BRCA* mutation. Of the 50 patients, 30 were identified as HRD-positive. Moreover, pCR and residual tumor burden (RCB 0/1) were both important indicators for tumor efficacy evaluation, of which pCR (RCB 0) was the main evaluation indicator.

Patients with HRD positivity were more likely to respond to standard NAC containing anthracyclines than HRD-negative patients, as indicated by a pCR (RCB 0) outcome (OR = 9.5, 95% CI 1.11–81.5, *p* = 0.040) (Table 6). Similar results were observed for the combined endpoint of RCB 0/1. In addition, patients with HRD positivity were more likely to achieve RCB 0/1 compared to non-deficient patients (OR = 10.29, 95% CI 2.02–52.36, *p* = 0.005) (Table 7). These results are applicable to a cohort of 35 patients without germline BRCA mutations. Patients with HRD-positive status showed a higher tendency towards an RCB 0/1 response than did HRD-negative patients (OR = 6.0, 95% CI 1.00–35.91, *p* = 0.050) (Table 7).

**Table 6:** Association of *BRCA* mutation and HRD status with pCR (RCB 0) from clinical cohort

All patients (*n* = 50)	pCR *n* (%)	Non- pCR *n* (%)	OR	95% CI	Logistic *P* value
*BRCA* status					
Non-mutated	5 (14)	30 (86)	1		
Mutated	6 (40)	9 (60)	4.0	0.99–16.24	0.052
HRD status					
Negative	1 (5)	19 (95)	1		
Positive	10 (33)	20 (67)	9.5	1.11–81.5	0.040
*BRCA* wild-type (*n* = 35)					
HRD negative	1 (5)	19 (95)	1		
HRD positive	4 (27)	11 (73)	6.91	0.68–69.86	0.102

HRD positive = HRD score ≥ 10 for AcornHRD or *BRCA* mutation

HRD negative = HRD score < 10 for AcornHRD

**Table 7:** Association of *BRCA* mutation and HRD status with RCB from clinical cohort

All patients (*n* = 50)	RCB0/1 n (%)	RCB2/3 n (%)	OR	95% CI	Logistic *P* value
*BRCA* status					
Non-mutated	8 (23)	27 (77)	1		
Mutated	10 (67)	5 (33)	6.75	1.78–25.58	0.005
HRD status					
Negative	2 (10)	18 (90)	1		
Positive	16 (53)	14 (47)	10.29	2.02–52.36	0.005
*BRCA* wild-type (*n* = 35)					
HRD negative	2 (10)	18 (90)	1		
HRD positive	6 (40)	9 (60)	6.0	1.00–35.91	0.050

HRD positive = HRD score ≥ 10 for AcornHRD or *BRCA* mutation

HRD negative = HRD score < 10 for AcornHRD

## Discussion

Genomic scar analysis is a very important HRD detection method. When non-homologous end joining (NHEJ) repair is initiated, it leaves “genomic scars”, which are traces of damage repair in the genome [34]. Cells with HRD cannot repair DNA double-strand breaks as effectively as cells with HR pathways. As a result, they exhibit genomic scarring, which refers to quantifiable genomic alterations that can be used to reverse the cell's HRD status. There are three main types of genomic scars caused by HRD: loss of heterozygosity (LOH), telomeric allelic imbalance (TAI), and large-scale state transitions (LST) [33]. To date, the Food and Drugs Administration has approved two products for clinical testing of HRD, namely Myriad myChoice ® CDx (myriad-oncology.com/mychoice-cdx) and FoundationFocus ™ CDx *BRCA* LOH (www.accessdata.fda.gov/cdrh_docs/pdf16/p160018c.pdf). Both products use the detection of *BRCA* gene mutations combined with the genomic scar to assess HRD status. The former contains the *BRCA* genes coding region and 54,091 single nucleotide polymorphisms (SNPs) population. The Genomic Instability Score (GIS) was obtained by comprehensively calculating three indicators: LOH, TAI, and LST, while GIS ≥ 42 was considered positive for genomic instability status [29, 35]. The latter calculated the proportion of fragments with LOH in this genome by covering 3500 SNPs in 324 genes on 22 chromosomes, and LOH accounted for ≥ 16%, that is, “high LOH [36]”. The above two commercial kits lack large-sample prospective clinical study data applied to the Chinese population; thus, studies promoting the development and clinical validation of these kits in China are warranted.

It has been confirmed that LST is feasible and has unique advantages for assessing HRD [24, 25, 33, 37]. LST is referred to the number of chromosomal breaks between flanking regions of at least 10 Mb [24, 38]. Moreover, it has been reported that the LST genomic signature accurately identified tumors with HRD and displayed excellent performance in a TNBC cohort, reaching almost 100% in sensitivity and specificity for HRD detection, where HRD was defined as *BRCA* inactivation [24, 25]. Furthermore, LST had better HRD evaluation performance in low-coverage sequencing than did LOH [26].

The ShallowHRD is a software tool based on mining copy number alterations profile from TCGA breast cancer that displays a high performance for HRD detection in breast cancers in low coverage genomic data [31]. Fundamental to evaluating the HRD status is the robust determination of copy number data, which can be obtained using either SNP arrays, whole exome sequencing (WES), or WGS. Comparing CNV derived from SNP array, WES, and WGS has revealed that WGS yields a more uniform distribution of quality parameters, such as genotype quality and coverage [39–41]. Moreover, studies indicated an excellent agreement (93.75%) between the original and down-sampled WGS-derived HR classification status [26]. WGS at low coverage robustly detects CNV, even in FFPE samples and liquid biopsies [42], at low cost and with easy-storable data outputs.

LCNA identified with Shallow whole-genome sequencing is increasingly popular in many diagnosis institutions. However, low-coverage sequencing also brings some challenges. Since the ShallowHRD data are based on Western cases, it is unclear whether it is applicable to Chinese patients [31]. In the initial use of ShallowHRD, it was found that its performance of *BRCA*-positive agreement rate in detecting HRD was poor. Furthermore, in the context of lower coverage genomes, the ability to accurately characterize somatic variations, including single-nucleotide variants (SNVs), breakpoints, and CNVs, is compromised, particularly in tumors with low cellularity or sequencing data exhibiting significant GC bias. Moreover, the uniformity of sequencing should be high; otherwise, it will be accompanied by serious noise pollution. The low-coverage sequencing has to be balanced with the sensitivity and uniformity for robustly calling somatic mutations.

To address the questions, we developed an algorithm named AcornHRD, which detects LCNA events based on a low-depth detection algorithm of ~ 1 × WGS reads. Compared with similar software ShallowHRD, AcornHRD achieved a good capacity for HRD detection and improved the obvious disadvantage of ShallowHRD of low *BRCA*-positive agreement rate in the standard and Chinese breast cancer cohort. Moreover, patients with high HRD score tumors evaluated by AcornHRD were significantly (*p* = 0.020) more likely to obtain RCB0/1 than those with low HRD score tumors; however, the same was not statistically significant by ShallowHRD. In summary, the performance of AcornHRD in evaluating HRD is superior to that of ShallowHRD. However, it is worth mentioning that different HRD assessment methods and their algorithms are not equivalent, and AcornHRD needs to be further compared with the two FDA-approved products.

Further, we investigated the relationship between the high HRD score and clinicopathological features of breast cancer. A high HRD score demonstrated a significant correlation with *BRCA* mutations, high Ki-67 expression, and a tendency towards ER negativity and PR negativity. Thus, the phenotype of high HRD score tumors is considered to be biologically aggressive. High HRD score was significantly more prevalent in the triple-negative breast cancer (TNBC) subtype than in the other three subtypes, which is consistent with previously reported results [25, 33, 43–45].

It has been shown that anthracycline-based regimens are effective in HER2-negative *BRCA*-mutated breast cancer [11, 13, 46–48]. Telli et al. [14] reported that a high HRD TNBC identified by next-generation sequencing was more sensitive to anthracyclines in the neoadjuvant setting. Recently, it has been reported that HRD tumors are more likely to benefit from anthracyclines, and HRD scores may be a clinically useful marker of chemosensitivity based on subtypes [45, 49]. In contrast, Imanishi et al. [43] reported the opposite result. We conducted a retrospective analysis to examine the relationship between HRD score and the response to neoadjuvant anthracycline therapy in HER2-negative breast cancer patients. The findings revealed a significant association between HRD score and RCB 0/I as well as pCR in the overall population cohort (*n* = 50). Similar outcomes were observed in the subset of patients without germline BRCA mutations (*n* = 35).

Previous studies have shown that response to neoadjuvant platinum-based therapy (pCR and RCB0/1) is significantly associated with HRD status in TNBC [11, 29, 50], suggesting the clinical utility of HRD scoring in selecting breast tumors that are more likely to respond to platinum-based regimens. Conversely, the GeparOLA study 50 demonstrated that neoadjuvant therapy with Olaparib resulted in a higher rate of pCR compared to a carboplatin-based regimen (55.1% vs. 48.6%) in patients with HER2-negative or TNBC and HRD. Consistent results were obtained in younger (< 40 years) and HR-positive patients (76.2% vs 45.5%, 52.6% vs 20.0%, respectively), suggesting that HRD may have a good application in predicting the efficacy of PARPi for neoadjuvant treatment.

This study has several limitations. These include a small sample size used for validation, which may limit generalizability. Additionally, the relationship between more HRR genes and HRD status was not discussed. While AcornHRD method demonstrates promising performance in HRD detection, further validation against established authoritative assays or algorithms is warranted. Future studies will focus on optimizing performance, validating in larger cohorts, analyzing more HRR genetic variants and improving clinical applicability. Despite these limitations, our AcornHRD method represents an advancement in HRD detection and offers valuable insights for improving patient care and treatment outcomes.

## Conclusions

In conclusion, we have devised AcornHRD, an HRD score algorithm that surpasses clinical variables or BRCA1/2 mutation status in effectively identifying tumors with a higher probability of responding to anthracycline-based neoadjuvant therapy in Chinese breast cancer patients. Moreover, AcornHRD holds potential for use in clinical applications and translational research, including patient screening for clinical trials and guiding the use of DNA-damaging drugs.

### Supplementary Information

Additional file 1.

## Data Availability

Research data are stored in an institutional repository and will be shared upon request to the corresponding author.
